# Levels of plasma 25-hydroxy vitamin D and risk of developing type 2 diabetes in a large Danish primary health care population

**DOI:** 10.1007/s00592-024-02368-0

**Published:** 2024-09-03

**Authors:** Cecilie Korneliusen Rohold, Henrik Løvendahl Jørgensen, Fie Juhl Vojdeman, Christian Medom Madsen, Anja Olsen, Anne-Marie Heegaard, Bent Struer Lind, Anne Tjønneland, Peter Schwarz, Peter Haulund Gæde

**Affiliations:** 1 University of Copenhagen, Blegdamsvej 3B, KBH N, 2200 Copenhagen, Denmark; 2 Department of Clinical Medicine, University of Copenhagen, Blegdamsvej 3B, KBH N,r, 2200 Copenhagen, Denmark; 3 Department of Clinical Biochemistry, Hvidovre Hospital, Kettegård Alle 30, 2650 Hvidovre, Denmark; 4 Department of Clinical Biochemistry, Holbæk Hospital, Smedelundsgade 60, 4300 Holbæk, Denmark; 5 Novo Nordisk A/S, Novo Alle 1, 2880 Bagsværd, Denmark; 6 Danish Cancer Institute, Strandboulevarden 49 KBH Ø, 2100 Copenhagen, Denmark; 7 Department of Drug Design and Pharmacology, University of Copenhagen, Jagtvej 60, KBH Ø, 2100 Copenhagen, Denmark; 8 Department of Endocrinology, Rigshospitalet, Blegdamsvej 9 KBH Ø, 2100 Copenhagen, Denmark; 9 Department of Endocrinology, Slagelse Hospital, Fælledvej 11, 4200 Slagelse, Denmark; 10 Faculty of Health Sciences, University of Southern Denmark, Winsløws Parken, J. B. Winsløws Vej 19, 3, 5000 Odense, Denmark

**Keywords:** Type 2 diabetes, Vitamin D, Primary health care, Hemoglobin A1c

## Abstract

**Aims:**

Plasma levels of Vitamin D (25(OH)D) have been suggested as a predictor for developing type 2 diabetes. The purpose of this study was therefore to investigate if a measurement of plasma 25(OH)D could predict the development of type 2 diabetes in a cohort of 222,311 individuals from primary healthcare in Denmark.

**Methods:**

The CopD-study database containing data from the Copenhagen General Practitioners Laboratory on blood tests conducted from April 2004 to January 2012 was used for identification of the study population. Incident type 2 diabetes was then defined as having at least two redeemed prescriptions of antidiabetics or at least two hospital contacts due to type 2 diabetes or one redeemed prescription and one hospital contact regarding type 2 diabetes.

**Results:**

A total of 222,311 individuals were included in the study, of whom 7652 (3.4%) developed type 2 diabetes during the follow-up period of minimum one year. Individuals who developed type 2 diabetes had a significantly lower median 25(OH)D level than persons in the non-diabetes group. The hazard ratio for development of type 2 diabetes increased by 15% per 10 n mol/L decrease in 25(OH)D level.

**Conclusion:**

In this study of 222,311 persons from primary health care in Denmark, we found a clear inverse relationship between 25(OH)D and the risk of developing type 2 diabetes.

Further studies should be conducted to clarify the mechanisms behind the relationship between 25(OH)D and type 2 diabetes and the effect of oral vitamin D supplementation on the development of type 2 diabetes.

## Introduction

Diabetes is an increasing global health challenge and about 462 million people worldwide suffer from type 2 diabetes [1]. Complications from having diabetes are serious and include peripheral vascular disease, neuropathy, and nephropathy [2]. Preventive measures are therefore needed for people at risk of developing diabetes. With this in view, it is important to identify possible risk factors for diabetes as this could help prevent future cases.

Vitamin D levels in plasma (25(OH)D) have been suggested as a risk factor for diabetes in several observational studies and meta-analyses [3–6]. However, most of these studies have been relatively small with less than 10,000 participants. Attempting to investigate the hypothesis of causality of the relationship between low vitamin D levels and incident type 2 diabetes, several mendelian randomization studies have been performed with some supporting this hypothesis and others not [7–10].

If a measurement of 25(OH)D could contribute to the identification of people at risk of developing diabetes, it would be possible to install preventive interventions such as exercise, improved diet and possibly, vitamin D supplementation. Earlier studies have shown that 25(OH)D might play a role in glucose metabolism via multiple pathways. 25(OH)D stimulates insulin secretion via multiple pathways including via the vitamin D receptor (VDR) in pancreatic cells and by regulation of calcium levels which in turn regulate insulin secretion [11, 12]. Moreover, vitamin D plays a role in the modulation of immune responses and in the lowering of systematic inflammation [13]. Other studies suggest that 25(OH)D and calcium deficiency have an effect on postprandial glycemia and insulin response and that oral vitamin D supplementation could help optimize these processes [14]. Besides its role in in calcium homeostasis, 25(OH)D has also been suggested to play an important role in various diseases such as cancer, cardiovascular disease, and immune dysfunction [15–17].

The effect of vitamin D supplementation has been analyzed in meta-analyses of randomized controlled trials. Thus, Li et al. found that oral vitamin D supplementation compared to placebo had an effect in enhancing serum 25(OH)D levels and reducing insulin resistance among type 2 diabetics [18], while Zhang et al. in another meta-analyses including persons with prediabetes showed that vitamin D supplementation reduces the risk of type 2 diabetes, although the benefit of the prevention of type 2 diabetes in this study could be limited to nonobese subjects [19]. Finally, a study by Mirhosseini et al. found that a minimum dose of 100 µg/d of vitamin D supplementation could reduce Hemoglobin A1c (HbA1c) levels [20]. Although much evidence thus already points toward the beneficial effect of vitamin D in type 2 diabetes, there are as yet, to our knowledge, no large observational studies with more than 100,000 participants. The purpose of this study was therefore to investigate if a measurement of plasma 25(OH)D could predict the later development of type 2 diabetes in a cohort of 222,311 individuals from primary healthcare in Denmark.

## Methods

**Fig. 1:**
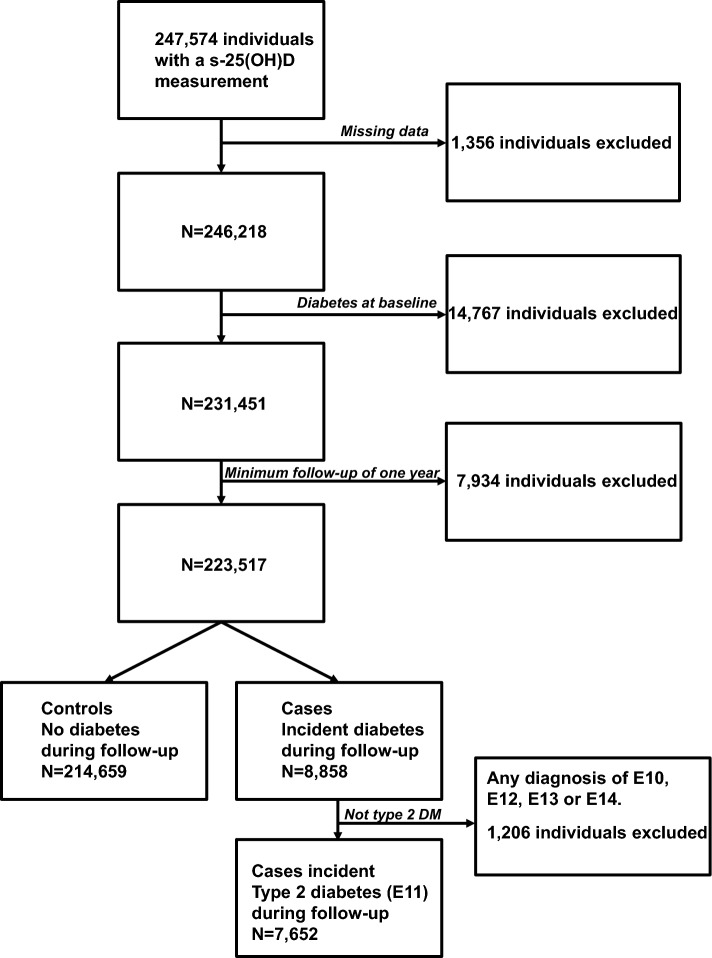
Flowchart of the selection process

The study population of 222,311 individuals was identified using the CopD-study (Copenhagen Vitamin D study) database which is a database containing data from the Copenhagen General Practitioners Laboratory. The database contains a wide range of blood tests conducted from April 2004 to January 2010 in the primary sector of the greater Copenhagen area. A total of 247,574 individuals had 25(OH)D analyzed. In this study, we excluded 1356 individuals due to missing data and 14,767 individuals diagnosed with diabetes at baseline. Furthermore, we excluded 7934 individuals due to lack of a minimum follow-up of one year. We thus included 223,517 individuals, of whom 214,659 were controls with no diabetes during follow-up and 8858 were incident diabetes cases during follow-up. Finally, we excluded 1206 individuals with other types of diabetes than type 2 diabetes, leaving 7652 cases of incident type 2 diabetes during follow-up, yielding a total study population of 222,311 individuals (Fig. 1).

In Denmark all citizens have an individual 10-digit civil registration number (CRN). The CRN allowed us to find information identifying diabetics in the Danish National Patient Registry (DNPR) [21]. The DNPR contains information about specific patient contacts with the Danish hospital services as well as diagnoses, treatments, surgical interventions, and examinations which are registered with the use of ICD10 (International Classification of Diseases 10th Revision) codes.

The Danish Register of Pharmaceutical Sales contains information on sale and deliveries of prescription medicine being sold in stores and at pharmacies but also on medicine dispensed by the physician or by hospitals in Denmark.

These registers were used to identify individuals for inclusion in our study. Incident diabetes was thus defined as having at least two redeemed prescriptions of antidiabetics, at least two hospital contacts due to diabetes (ICD10 codes being E10, E11, E12, E13; E14) or one redeemed prescription (each type of ATC code A10) and one hospital contact regarding diabetes. Incident type 2 diabetes was subsequently identified by excluding persons having at least one other diabetes diagnosis code not being E11.

Women between 18- and 40-years were excluded if their only antidiabetic prescriptions were metformin to exclude cases of polycystic ovary syndrome (PCOS). Simultaneously, cases with PCOS diagnoses in the Danish National Patient Registry were excluded. Prescriptions or hospital contacts one year after diagnosis of gestational diabetes were also excluded.

The plasma level of 25(OH)D was assessed as previously described [22].

## Approvals

The study was approved by the Danish Health and Medicines Authority (file number: 3–3013-858/1/), the ethical committee of The Capital Region of Denmark (protocol number: H4-2014-FSP) and by the Danish National Data Protection Agency (file number: 2012–41-0390).

## Statistics

Differences between continuous variables were tested using Mann Whitney U tests while differences in the distribution of categorical variables were tested using Chi-square tests.

In order to investigate the shape of the relationship between incident type 2 diabetes and 25(OH)D, we divided 25(OH)D into 12 groups from 10 nmol/L to 120 mmol/L, by rounding the plasma 25(OH)D measurement to the nearest 10 nmol/L. The 120 mmol/L group includes all results higher than 120 nmol/L. The number of incident cases of type 2 diabetes per 100,000 individuals for each of the 12 groups was plotted against the group vitamin D level. A second-degree polynomial was found to be the best fit to the data.

Proportional hazards regression analyses were used to investigate the relationship of incident type 2 diabetes and plasma 25(OH)D. The nonlinear relationship between incident type 2 diabetes and 25(OH)D was analyzed using a Cox proportional hazards model, where 25(OH)D was entered in the model as a restricted cubic spline with five knots placed at the 5th, 25th, 50th, 75th and 95th percentiles of the 25(OH)D concentration. A 25(OH)D level of 50 nmol/L was used as reference. In the cox regression analyses, events or censoring were assessed between June 2005 and December 2014. The follow-up period between the measurement of 25(OH)D and time to event/censoring had to be at least one year. The events were a diagnosis of type 2 diabetes, while individuals who died, emigrated or reached end-of-study (31 December 2014) were censored. In both analyses, adjustment was made for age, sex, income and Charlson Comorbidity Index (CCI).

All analyses were performed using SAS version 9.4 (SAS Institute, Cary, NC, USA). For all the statistical analyses, *p* < 0.05 was considered statistically significant.

The data used in this study is anonymized and deposited at Statistics Denmark (ref no 706282), accessible via a secure access provided by Statistics Denmark.

## Results

A total of 222,311 individuals were included in the study, of whom 7652 (3.4%) developed type 2 diabetes during the follow up period. In the type 2 diabetes group, 57.7% were female compared to 66.8% in the non-diabetes group. The mean age was 57.8 years in the type 2 diabetes group compared to 48.7 years in the non-diabetes group. In the type 2 diabetes group, 1.8% had a CCI score of ≥ 4 compared to 1.1% in the non-diabetes group. In individuals who developed type 2 diabetes, the median 25(OH)D level was 37 nmol/L (IQR 22–56) compared to 48 nmol/L (IQR 29–69) in the non-diabetes group. The follow-up period was 3.6 years for individuals who developed type 2 diabetes during the follow-up and 6.1 years for the non-diabetes group (Table 1).

**Table 1:** Basic characteristics, values shown are median (interquartile range) for continuous values and number (percent) for categorical data

	DM2 during follow-up	No DM2 during follow-up	*P*
N	7652	214,659	–
Age (years)	57.8 (47.5;67.4)	48.7 (33.3;64.5)	< 0.0001
Follow-up (years)	3.6 (2.3; 5.2)	6.1 (5.4; 6.9)	< 0.0001
Income (1000 DKR)	171 (123; 287)	206 (130; 328)	< 0.0001
Females N (%)	4412 (57.7)	143,385 (66.8)	< 0.0001
Males N (%)	3240 (42.3)	71,274 (33.2)	–
s-25(OH)D (nmol/l)	37 (22;57)	48 (29;69)	< 0.0001
CCI			
0	5267 (68.8)	170,605 (79.5)	< 0.0001
1	1423 (18.6)	25,670 (12.0)	
2	596 (7.8)	12,140 (5.7)	
3	228 (3.0)	3765 (1.7)	
> = 4	138 (1.8)	2479 (1.1)	

The difference in follow-up period is caused by the follow-up period of the DM2 group ending at the time of DM2 diagnosis whereas the no DM2 group was followed up until the end-of-study, death or emigration Age at the time of the 25(OH)D measurement and sex were calculated using the civil registration number. The time and the result of the P-25(OH)D measurement were extracted from the laboratory information system at the Copenhagen General Practitioners Laboratory. All other data was extracted from national registers

*CCI* Charlson comorbidity index, *DM2* type-2-diabetes

**Fig. 2:**
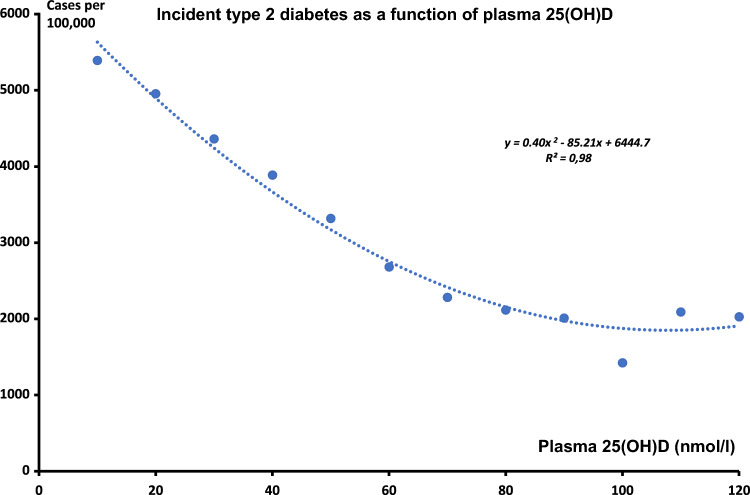
Incident type 2 diabetes as a function of 25(OH)D in nmol/L rounded to the nearest 10 nmol/L. The 120 mmol/L group includes all results higher than 120 nmol/L

**Fig. 3:**
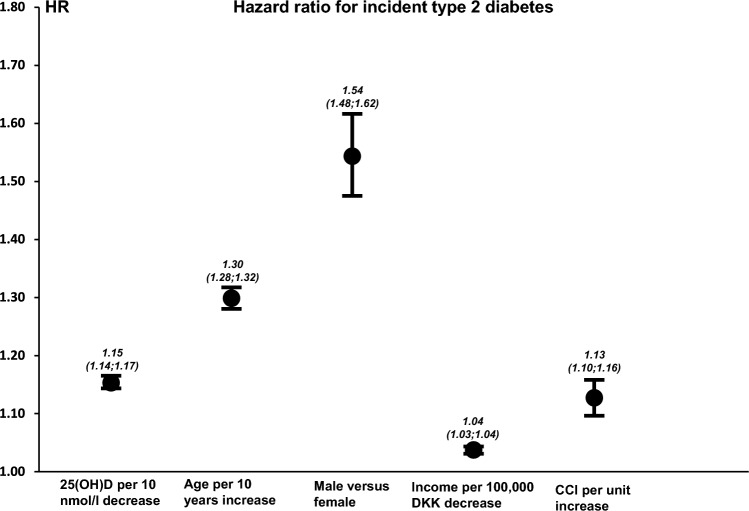
Hazard ratio for incident type 2 diabetes adjusted for age, sex, income and Charlson Comorbidity Index (CCI)

Unadjusted correlation analysis showed a non-linear relationship between 25(OH)D levels and incident type 2 diabetes. Low levels of 25(OH)D correlated with a higher number of incident type 2 diabetes while higher levels of 25(OH)D correlated with a lower number of incident diabetes (Fig. 2). In a multivariate cox regression analysis adjusted for for age, sex, income and CCI, the hazard ratio for incident type 2 diabetes was 1.15 (95% CI 1.14;1.17) per 10 nmol/L decrease of 25(OH)D levels. When looking at the co-variates in the model, males compared to females had a hazard ratio of 1.54 (95% CI 1.48; 1.62) for developing type 2 diabetes. Per 10 years increase in age, the hazard ratio for developing diabetes was 1.30 (95% CI 1.28; 1.32). For income per 100,000 DKK decrease, the hazard ratio was 1.04 (95% CI 1.03; 1.04). CCI per unit increase had a hazard ratio of 1.13 (95% 1.10; 1.16) (Fig. 3).

**Fig. 4:**
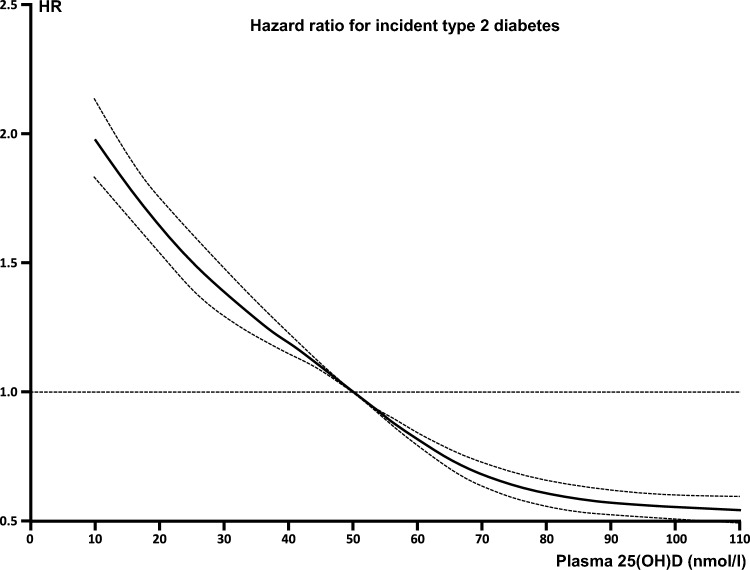
Hazard ratio for incident type 2 diabetes adjusted for age, sex, income, and Charlson Comorbidity Index (CCI) with 25(OH)D entered into the model as a restricted cubic spline with five knots placed at the 5th, 25th, 50th, 75th and 95th percentiles of the 25(OH)D concentration. A 25(OH)D level of 50 nmol/Liter was used as reference. The dotted lines represent 95% confidence intervals

Using restricted cubic spline Cox regression analysis with estimates adjusted for age, sex, income, and CCI, we found a similar nonlinear inverse relationship between 25(OH)D levels and hazard ratio for incident type 2 diabetes. Compared to a 25(OH)D level of 50 nmol/L, a 25(OH)D level of approximately 10 nmol/L increased the hazard ratio to approximately 2.0 (95% CI 1.8;2.2). Conversely, a 25(OH)D level of approximately 100 nmol/L decreased the hazard ratio to approximately 0.55 (95% CI 0.5; 0.6) compared to a 25(OH)D level of 50 nmol/L (Fig. 4).

## Discussion

In this study of the relationship between plasma 25(OH)D levels and the risk of developing type 2 diabetes in 222,311 individuals, we found an incidence of type 2 diabetes of 3.4% during a follow-up period of minimum one year. Individuals who developed type 2 diabetes had a significantly lower median 25(OH)D level than those in the non-diabetes group. The hazard ratio for development of type 2 diabetes increased by 15% per 10 nmol/ decrease in 25(OH)D level. In addition, we found that increased age, male sex, decreased income and increased CCI score all increased the risk of incident type 2 diabetes in accordance with previous knowledge [23].

These results are in accordance with earlier studies. A study by Song et al. also found a relationship between 25(OH)D levels and developing type 2 diabetes. They found that 25(OH)D levels ≥ 0 nmol/L were associated with a lower risk of type 2 diabetes similarly to our study [24].

Likewise, a systematic review and meta-analysis by Forouhi et al. found an inverse relationship between 25(OH)D concentration and type 2 diabetes [25].

On the other hand, a study by Grimnes et al. found no relationship between serum 25(OH)D and type 2 diabetes in a population-based 11-year follow-up study after adjusting for BMI [26].

Furthermore, a cohort study by Husemoen et al. found low serum 25(OH)D to be associated with type 2 diabetes but only in overweight-obese and not in normal weight individuals [27].

Vitamin D deficiency is potentially preventable and a closer focus on low levels of 25(OH)D could therefore play an important role in the prevention of developing type 2 diabetes.

A systemic review and meta-analysis by Li et al., looking at the effects of oral Vitamin D supplementation on glycemic control, found that among type 2 diabetics, oral Vitamin D supplementation reduced insulin resistance compared to placebo. In contrast, they found no benefits in terms of improving fasting blood glucose, HbA1c or fasting serum insulin levels [18].

In a meta-analysis from 2020, Barbarawi et al. found that Vitamin D supplementation of ≥ 1000 IU/day resulted in a significantly lower risk of type 2 diabetes in individuals with prediabetes [28].

On the other hand, a randomized placebo-controlled trial by Pittas et al. found that a daily vitamin D_3_ supplementation did not result in a significantly lower risk of diabetes compared to placebo [29].

As we found that low 25(OH)D levels are associated with increased risk of developing type 2 diabetes, individuals experiencing low levels of 25(OH)D might benefit from diabetes or prediabetes screening. In this way 25(OH)D could function as a marker for individuals at risk of developing type 2 diabetes. Early intervention in individuals with prediabetes could possibly prevent or delay the development of type 2 diabetes and subsequent diabetic complications [30].

### Strengths and limitations

The major strength of this study was the very large sample size which allowed us to include a high number of individuals in this study. It was possible to identify the study population of 222,311 individuals due to the completeness and reliability [21] of the Danish registers combined with the civil registration numbers.

Observational studies such as the present study should be interpreted with caution because they cannot prove causality, and reverse causation may explain the observed associations, and unknown lifestyle factors related to less sunlight exposure or unhealthy diet may be the driving factors explaining the link between vitamin D deficiency and incident type 2 diabetes. Furthermore, the individuals in the present study went to their general practitioner for reasons we do not know and in that sense, they do not represent a sample of the general population. Nevertheless, it is a very large sample of almost 25 percent of the inhabitants in the catchment area of the Copenhagen General Practitioners Laboratory. Furthermore, the number of vitamin D measurements in the study period increased almost exponentially which could indicate that the requisition of this test was added as an extra feature, not necessarily related to the reason for going to the general practitioner.

Unfortunately, we did not have data on anthropometry, like BMI, information on dietary habits, information on family history of diabetes or the level of physical activity which could all influence 25(OH)D levels and which have been shown to be associated with the development of type 2 diabetes [23].

By far, the most important limitation is the lack of data on BMI, since obesity is the strongest risk factor for type 2 diabetes [23]. Combined with the fact that numerous studies have confirmed the correlation between obesity and vitamin D deficiency [31], the effect of low vitamin D levels on incident type 2 diabetes in the present study would most likely have been attenuated if adjusted for BMI.

Finally, the study could not examine the potential role of oral vitamin D supplementation in the prevention of type 2 diabetes since these supplements are not on prescription and therefore not registered in The Danish Register of Pharmaceutical Sales.

## Conclusion

In this study of 222,311 persons from primary health care in Denmark, we found a clear inverse relationship between 25(OH)D and the risk of developing type 2 diabetes.

Further studies should be conducted to clarify the mechanisms behind the relationship between 25(OH)D and type 2 diabetes and the effect of oral vitamin D supplementation on the development of type 2 diabetes.

## Data Availability

For regulatory reasons, the source data for this study is not publicly available.
